# Effect of heat stress on reproductive performances of dairy cattle and buffaloes: A review

**DOI:** 10.14202/vetworld.2016.235-244

**Published:** 2016-03-05

**Authors:** Soumya Dash, A. K. Chakravarty, Avtar Singh, Arpan Upadhyay, Manvendra Singh, Saleem Yousuf

**Affiliations:** Dairy Cattle Breeding Division, ICAR-National Dairy Research Institute, Karnal, Haryana, India

**Keywords:** buffaloes, cattle, heat stress zone, reproductive traits, temperature humidity index

## Abstract

Heat stress has adverse effects on the reproductive performances of dairy cattle and buffaloes. The dairy sector is a more vulnerable to global warming and climate change. The temperature humidity index (THI) is the widely used index to measure the magnitude of heat stress in animals. The objective of this paper was to assess the decline in performances of reproductive traits such as service period, conception rate and pregnancy rate of dairy cattle and buffaloes with respect to increase in THI. The review stated that service period in cattle is affected by season of calving for which cows calved in summer had the longest service period. The conception rate and pregnancy rate in dairy cattle were found decreased above THI 72 while a significant decline in reproductive performances of buffaloes was observed above threshold THI 75. The non-heat stress zone (HSZ) (October to March) is favorable for optimum reproductive performance, while fertility is depressed in HSZ (April to September) and critical HSZ (CHSZ) (May and June). Heat stress in animals has been associated with reduced fertility through its deleterious impact on oocyte maturation and early embryo development. The management strategies *viz*., nutrition modification, environment modification and timed artificial insemination protocol are to be strictly operated to ameliorate the adverse effects of heat stress in cattle and buffaloes during CHSZ to improve their fertility. The identification of genes associated with heat tolerance, its incorporation into breeding program and the inclusion of THI covariate effects in selection index should be targeted for genetic evaluation of dairy animals in the hot climate.

## Introduction

The cattle and buffaloes are known for their milk production and they contribute approximately 96% to total milk production in India. Though milk production in India has been reached to 132.4 million tonnes in 2012-13 with a growth rate of 3.5%, but there is high demand of milk [1] and it is projected that by 2030 India will be able to produce 200 million tonnes of milk [2]. This target will be achieved if there is the optimum balance between productivity and fertility. Fertility is a very broad term which is influenced by various factors including genetic, nutritional, hormonal, physiopathology, management and environment or climate. The fertility traits in dairy animals show a very low heritability value, and this indicates that most of the variations in the fertility are determined by non-genetic factors or environmental effects [3]. The main natural physical environmental factors affecting livestock system includes air temperature, relative humidity (RH), solar radiation, atmospheric pressure and wind speed (WS) [4]. All these environmental factors are pooled to produce heat stress on animals, which is defined as any combination of environmental variables producing conditions that are higher than the temperature range of the animal’s thermoneutral zone (TNZ) [5]. Heat stress has an adverse effect on reproduction traits of dairy cattle [6,7] and buffaloes [8]. The negative influence of heat stress on reproduction traits of cattle and buffaloes can be quantified through formulating temperature humidity index (THI). The THI is a single value which incorporates the both of the air temperature and RH in the index [9]. Heat load index (HLI) is another index to measure the level of heat stress in feedlot cattle through incorporating the RH, wind speed and black-globe temperature (BGT) [10]. A negative correlation exists between reproduction traits of cattle and buffaloes with THI and animals experience the adverse effects of heat stress when the THI crosses a threshold level. The conception rate in lactating dairy cows declines with THI more than 72-73 in cattle [11,12] and 75 in buffalo [13]. The significant (p≤0.05) decline in the first service pregnancy rate of dairy cattle was observed at THI level above 72 [14] and heat stress was one of the major factors for a significant reduction in a pregnancy rate of crossbred cows in India [15]. The buffaloes are also susceptible to heat stress with respect to decline in fertility above THI level 75 in a subtropical climate [8]. This review was aimed to determine the influence of heat stress in relation with THI on reproductive performances of cattle and buffaloes.

## Global Warming and its Impact on Animal Reproduction

Global warming has a great impact on the reproductive activity of cattle and buffaloes. Global warming has risen the surface temperature about 0.7°C since the early 20^th^ century. It is anticipated that the temperature rise will be 1.8-4°C by 2100 [16]. The Intergovernmental Panel on Climate Change also indicated that the developing countries tend to be more vulnerable to extreme climatic events as they largely depend on climate sensitive sectors like agriculture and forestry. The greenhouse gas emission from agriculture sector is the most important factor for global warming, and livestock sector share 18% of total greenhouse gas emissions. The productive and reproductive performances of cattle and buffaloes are likely to be aggravated due to climate change and global warming. Assessment of the potential direct impacts of climate change on the reproduction of buffaloes indicate that there is increasing trend in incidences of silent estrus, the decline in reproductive activity and conception of buffaloes due to increase in air temperature during summer [17-19].

## Different Heat Stress Models for Formulating THI and HLI

Hahn *et al*. [4] demonstrated the main natural physical environmental factors affecting livestock system includes air temperature, RH, WS, solar radiation, precipitation, atmospheric pressure, ultraviolet light and dust. This leads to the establishment of thermal indices which can better reflect the thermal stress of the animal. Hence, a variety of indices is used to estimate the degree of heat stress affecting performance traits *viz*., production traits, reproduction traits and growth traits in cattle and buffaloes. The most common among these indices is the THI. A number of methods have been developed over the years to formulate the THI, which is applied to measure the level of heat stress on animals (Table-1). The THI is the common measure of heat stress for humans through combining the dry bulb and wet bulb temperature [9]. Thereafter, formulas for calculation of THI were extended by including RH or dew point temperature [20,21].

**Table-1:** Different heat stress models for formulating temperature humidity indices.

Heat stress models	Formulae	References
THI 1	[0.4×(T_db_+T_wb_)]×1.8+32+15	[9]
THI 2	(0.35×T_db_+0.65×T_wb_)×1.8+32	[22]
THI 3	(T_db_+T_wb_)×0.72+40.6	[20]
THI 4	(1.8×T_db_+32)−(0.55−0.0055×RH)×(1.8×T_db_−26)	[20]
THI 5	(0.55×T_db_+0.2×T_dp_)×1.8+32+17.5	[20]
THI 6	T_db_+(0.36×T_dp_)+41.2	[21]
THI 7	(0.8×T_db_)+[(RH/100)×(T_db_−14.4)]+46.4	[23]

T_db_=Dry bulb temperature, T_wb_=Wet bulb temperature, RH=Relative humidity, THI=Temperature humidity index

**Figure-1:**
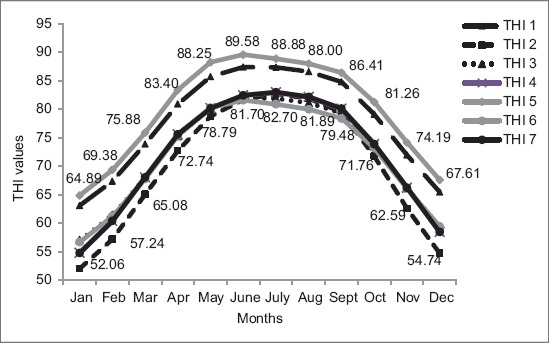
Monthly average temperature humidity index (THI) values with seven different THI models. Data are from Dash *et al*. [24].

All these THI models were used in a study conducted by Dash *et al*. [24] at Karnal in India. Month wise average THI values with seven different THI models during 20 years from 1993 to 2012 were presented in Figure-1. The maximum THI values were observed in the month of June as 87.41, 81.90, 89.58 and 81.60 with THI model 1, 3, 5 and 6, respectively. For other THI models (2, 4 and 7), maximum THI values were found in the month of July as 82.70, 82.97 and 82.99, respectively. The minimum THI values were found in the month of January for all the THI models (1-7) as 63.12, 52.06, 57.07, 54.82, 64.89, 56.71 and 54.80, respectively. After a thorough analysis of all the seven THI models with pregnancy rate of Murrah buffaloes, the THI model 1 [(0.4 × (T_db_ + T_wb_)] × 1.8 + 32 + 15) was identified as the best THI model for studying the effects of heat stress on pregnancy rate of buffaloes [24]. Bohmanova *et al*. [25] compared all the seven THI models and drawn the conclusion that there is variation in use of THIs according to the climatic condition. The THI which put more weight on the humidity is more appropriate for humid climates, whereas the indices with the more weight on ambient temperature work best under semiarid climates.

Various modifications to the THI have been proposed through formulating the HLI. Gaughan *et al*. [10] developed the HLI based on the weather parameters *viz*., RH, WS and BGT in feedlot cattle during hot weather. The HLI consists of two parts based on BGT threshold of 25°C as follows:

HLI_BGT>25_ = 8.62 + (0.38 × RH) + (1.55 × BGT) – (0.5 × WS) + e (2.4 − WS)

Where e is the base of the natural logarithm

HLI_BGT<25_ = 10.66 + (0.28 × RH) + (1.3 × BGT) − WS

The THI and HLI are considered as the appropriate guides to quantify the heat stress on animals. When an animal is exposed to THI or HLI above the threshold, then the core body temperature increases and the longer the duration of exposure above the threshold, the greater is the heat stress to animals.

### Thermo-neutral zone (TNZ) and heat stress zone (HSZ)

**Figure-2:**
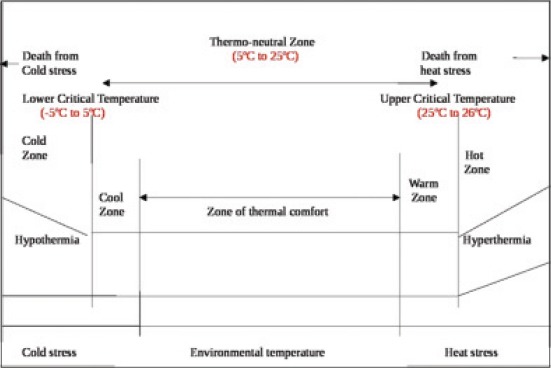
Thermoneutral zone in cattle.

The environmental stress is aggravated due to global warming accompanied with periods of extreme weather. If there are high temperature and humidity in the environment, then it is very difficult for the animal to dissipate heat and the animal undergoes heat stress. Heat stress is the state at which mechanisms get activated to maintain an animal’s body thermal balance when exposed to uncomfortable elevated temperature. Heat stress is defined as any combination of environmental parameters producing conditions that are higher than the temperature range of the animal’s TNZ [5]. The TNZ explains about the inter-relationship between the animal and the environment and it is defined as the range within which metabolic rate is minimal, and a healthy animal can make physical adaptation to maintain the normal body temperature with minimal change in metabolic activity. In general, the TNZ is surrounded by lower critical temperature and higher critical temperature (Figure-2). The upper critical temperature has been defined in dairy cows as 25-26°C [26].

When the environmental temperature moves away from the upper critical temperature, the detrimental effects of heat stress in terms of reduction of milk production, changes in milk composition and lower reproductive performances are observed in cattle and buffaloes. Various authors developed different zones whether the animals are comfortable or susceptible to heat stress based on the THI values.

#### Dairy cattle

Armstrong [27] categorized THI values into five different classes as no stress with THI value <72, mild stress (72-78), moderate stress (79-88), severe stress (89-98) and dead cows with THI >98 (Table-2). Similarly, The Livestock Weather Safety Index quantified environmental conditions using the THI as formulated by Thom [9] and it described the HSZ into four categories with different range of THI under each category like normal (≤74), alert (74< THI <79), danger stress (79≤ THI <84) and emergency stress (THI ≥84) in livestock. McDowell *et al*. [28] developed three different classes of THI as comfortable (≤70), stressful (71-78) and extreme distress (>78). Moran [29] described five categories of THI as no stress (<72), mild stress (72-78), severe stress (78-89), very severe stress (89-98) and dead cows (>98). The importance of classifying the THI into different classes involves the determination comfortable zone or HSZ where the animals have been exposed to heat stress. The acute exposure to extreme heat load is associated with disturbance to a physiological mechanism to the body like rapid respiration and excessive saliva production along with significant depression in reproductive performances in animals [30].

**Table-2:** Classification of zones based on THI values in cattle with THI model [27].

THI	Stress level	Symptoms in cattle	Symptoms in buffalo
<72	None	Optimum productive and reproductive performance	Optimum productive and reproductive performance
72-78	Mild	Dairy cows seek for shade, increase in respiration rate and dilation of blood vessels	Elevation in rectal temperature and respiration rate
79-88	Moderate	Increase in respiration rate and saliva secretion. Reduction in feed intake and water consumption. Body temperature is increased and reproductive performances are severely affected in cattle	Respiration rate is significantly increased. Dry matter intake of buffalo is decreased and ratio of forage to concentrate intake is decreased. Water intake in buffalo is significantly increased
89-98	Severe	There is rapid increase in respiration and excessive saliva production. The reproductive performances in animals are significantly decreased	Excessive panting and restlessness are observed. Rumination and urination are lowered along with a negative impact on reproductive performances in buffaloes
>98	Danger	Heat stress is extreme and cows may die	Heat stress is extreme and buffaloes may die

THI=Temperature humidity index

#### Buffalo

Dash *et al*. [31] studied the reproductive performance of Murrah buffaloes in relation with THI values and identified three different zones as non-HSZ (NHSZ), HSZ and critical HSZ (CHSZ). The months from October to March were included under NHSZ with THI values 56.71-73.21 and months from April to September were incorporated under HSZ with THI values 75.39-81.60 while the months of May and June were identified as the CHSZ within the HSZ with THI values 80.27-81.60 (Table-3).

**Table-3:** Classification of zones based on THI values in buffalo [31].

Zones	Months	THI	

		Average	Range
NHSZ	October, November, December, January, February, March	64.08	56.71-73.21
HSZ	April, May, June, July, August, September	79.42	75.39-81.60
CHSZ	May, June	80.83	80.27-81.60

NHSZ=Non-heat stress zone, CHSZ=Critical heat stress zone, HSZ=Heat stress zone, THI=Temperature humidity index

#### Effect of heat stress on reproductive performances of cattle and buffaloes

The productive and reproductive performances of dairy cattle and buffaloes varied according to the prevailing climate of their inhabitation. The major climatic zones include tropical, subtropical and temperate. The temperate zone is the most ideal for higher productivity in dairy animals. Therefore, this review focused the effect of heat stress on different fertility traits of cattle and buffaloes *viz*., service period, conception rate and pregnancy rate in tropical/subtropical as well as temperate climate.

### Effect of heat stress on service period in tropical or subtropical climate

#### Dairy cattle

The heat stress is considered as one of the main factors affecting reproductive performances in dairy cattle. High temperature in summer months combined with a high level of RH has adverse effects on reproductive performance in cows. In Thailand, the lowest THI of 72 was observed in December and highest mean THI was observed in April as 80. The cows which calved in February had the longest service period as 299±11 days while cows that calved in the months of October or November had a significantly shorter mean service reported as 133±7 days [32]. Boonkum *et al*. [33] also found the greatest service period of 154-day in Thai crossbred Holstein cows for calving in March and the lowest service period of 128-day for calving in the month of October. In summary, calving month greatly affects the phenotypic variation in service period of Thai Holstein crossbred cattle. The season of calving had highly significant (p<0.01) effect on service period of Sahiwal cattle. The cows calved during summer months had longest service period of 159±3 days, whereas autumn calvers had the shortest service period of 148±4 days [34]. Heat stress controls the mechanism of hypothalamic-hypophyseal-ovarian axis in animals. The heat stress causes hyper-prolactinemia, reduction in luteinizing hormone frequency, poor follicle maturation and decreased oestradiol production leading to ovarian inactivity in cattle [35].

### Effect of heat stress on service period in temperate climate

The non-return rate (NR) is currently used in as a measure of reproductive ability in cattle. When the cow does not return to further insemination in the same lactation, then the success in conception is assumed. Ravagnolo and Misztal [6] reported that NR45 of Holstein cattle showed a decrease rate of 0.005 per unit increase in THI on the day of insemination at THI >68 and NR45 was significantly lower and more susceptible to elevation in THI for first parity cows than second, third and fourth lactation cows (0.008 vs. 0.005 decrease). The seasonal trend for service period is observed in cattle. The service period in Holstein cows was longest (166 days) for March/April calving and shortest (130 days) for September calving [36]. The conclusion is drawn from the above research is that the service period of cows was longest for spring and shortest for fall calvings. High temperature combined with a high level of humidity in spring and summer season results in physiological disorders, affecting the digestive system, acid-base chemistry, blood hormones and finally resulting in longer service period in cows.

#### Buffalo

The month wise average service period of Murrah buffaloes was analyzed with the monthly average THI values [13]. The average service period of Murrah buffaloes was prolonged (180 days) in the month May with the corresponding THI value of 80.27. On the contrary, the lowest average service period (119 days) was observed at average THI 67.80 in the month of March. The service period of buffaloes was found increased with increase in average THI above 75 [13]. The cooler months with lower THI values caused decrease in service period while the months with higher THI values above threshold level 75 were associated with increase in service period in Murrah buffaloes.

### Effect of heat stress on conception rate in tropical or subtropical climate

#### Dairy cattle

A significant change in conception rate of cattle was observed in response to the major climatic variables like temperature and humidity, which are combined to form the THI and the THI is popularly used to assess the fertility in bovines. Lactating dairy cows are particularly sensitive to heat stress because of high metabolic heat production inside the body associated with increased milk production. The negative effects of heat stress on conception rate of cattle are most evident when THI level crosses a threshold level. When the THI on the day of service is more than 72, it decreases the conception rate of dairy cows in Australia. The high heat load 3-5 weeks before service and 1 week after service was associated with reduced conception rate in cattle [11].

### Effect of heat stress on conception rate in temperate climate

The conception rate of lactating dairy cows is highly affected by heat stress. Garcia-Ispierto *et al*. [7] reported that negative effects of heat stress on conception rate of Holstein cows seem to appear when THI ≥75 on 3 days prior to artificial insemination and the effects of heat stress are more evident in the form of declining conception rate from 30.6% to 23% when THI was above 80 in North-Eastern Spain. The threshold THI for the influence heat stress on conception rate of lactating dairy cows in Germany was 73 and the greatest negative impact of heat stress on conception rate was observed 21-1 day before breeding [12]. The conception rate in Holstein cows was found lowered following services performed in hot months. Nabenishi *et al*. [37] observed that conception rate of lactating dairy cows during the hot period (July to September) was significantly lower (p<0.01) with 29.5% as compared to the conception rate of 38.2% during the cool period (October to June). The reductions in conception rates in hot periods are due to the combined effects of environmental heat, which produces an alteration in the synthesis of reproductive hormones [4]. The heat stress during the summer season is able to change the follicular microenvironment of highly productive dairy cows, and the detrimental effect of heat stress is associated with physiological processes of the establishment and maintenance of pregnancy after fertilization [35].

#### Buffalo

The incidence of seasonal reproductive behavior is a more common in buffaloes. Buffaloes are sexually activated by decreased day length and temperature [38]. The highest breeding frequency in buffaloes is found during the winter and the lowest in the summer season. Abayawansa *et al*. [39] reported that maximum percentage of buffaloes exhibited postpartum estrous during the month of September followed by October and minimum during April and May due to high maximum air temperature. Silent estrus is the most important limiting factor especially during hot months which leads to poor reproductive efficiency in buffaloes [40]. Dash [13] studied in detail regarding the monthly average conception rate of Murrah buffaloes with monthly average THI values over a year. The highest average conception rate was observed as 78% in the month of October while the lowest was 59% in the month of August. The threshold THI for conception rate was identified as 75 for the reason that with increase in average THI above threshold 75, the decline in overall conception rate was observed in Murrah buffaloes [13]. Heat stress results in a significant reduction in conception rate during the hot and humid-hot months when the monthly average THI is higher than 75 in buffaloes.

### Effect of heat stress on pregnancy rate in tropical or subtropical climate

#### Dairy cattle

Pregnancy rate is defined as the percentage of non-pregnant cows that become pregnant during each 21-day period. Now the pregnancy rate is more preferred compared to service period as an indicator of reproductive success because pregnancy rate can be easily defined and available soon. The pregnancy rate of animals is declined with respect to increase in THI above a threshold level. McGown *et al*. [14] reported that an increase in THI above 72 which corresponds to temperature 25°C and RH 50% resulted in a significant decrease in the first service pregnancy rate of Holstein cattle in Queensland, Australia. Another finding indicates that the month of insemination significantly (p<0.05) influenced the pregnancy rate in Holstein cows. A substantial decline in pregnancy rate was observed from 34.1% to 15.7% with increase in mean THI from 69 in May to 74 in July in a subtropical climate of Egypt [41]. The conception and the pregnancy rate of the Holstein dairy cattle were negatively affected by higher THI level under Egyptian subtropical conditions. The conception and the pregnancy rate of the Holstein cattle decreased from 35.8% and 29.4%, respectively, at low THI (<THI 70) to 16.1% and 12.1%, respectively, at high THI level of 80-85 [42]. The significant (p<0.05) reduction in a pregnancy rate of crossbred dairy cattle due to heat stress was also evident in India. When the dairy cattle was in TNZ, the pregnancy rate of was estimated as 32.6%, but it was significantly decreased to 20.5% when the animals came into HSZ [15]. In summer, risks for ovulatory failure, impaired oocyte quality or embryonic development, reduced progesterone production and increased embryo mortality may be the possible reasons for dramatic decline in fertility in animals [35].

### Effect of heat stress on pregnancy rate in temperate climate

The highest pregnancy rate of Holstein-Friesian dairy cattle was observed in September-November as 32% while the lowest pregnancy rate of 24% in March-May in South-Eastern United States [43]. The lower pregnancy rate is due to the delay of rebreeding cows in the summer months with a high level of heat stress. Amundson *et al*. [44] observed the negative associations of THI with pregnancy rate of *Bos taurus* crossbred cows in all three breeding seasons from 0 to 21 days, 0 to 42 days, and 0 to 60 days, respectively. However, the change was more pronounced during the first 21 days of the breeding season with a −2.06% change in pregnancy rate for each unit of change in THI value.

#### Buffalo

The THIs play an important role in the reproductive functions of buffaloes and it is suggested that THI >75 has a negative effect on reproductive performances of buffaloes in the tropical areas of Amazon basin in Brazil [45]. In a similar manner, a distinct relationship was observed between THI and pregnancy rate in Murrah buffaloes. The average pregnancy rate of Murrah buffaloes was found declining from 0.41 to 0.25 with the onset of THI ≥75 [8]. When the monthly average pregnancy rate of Murrah buffaloes was analyzed with monthly average THI values, then the lowest average pregnancy rate was observed as 0.25 in the month of July with corresponding THI 80.88 and the highest average pregnancy rate was obtained as 0.58 in the month of November at average THI value 66.09 [8]. The threshold THI for pregnancy rate in buffaloes was determined as 75 above which the detrimental effects of heat stress are affecting fertility are observed in buffaloes.

### Impact of heat stress on oocyte and embryo quality

Heat stress has adverse effects on reproductive performances of cattle and buffaloes. The Higher ambient temperature during the summer has been associated with reduced fertility in dairy cattle through its deleterious impact on oocyte maturation and early embryo development [35]. There are several possible mechanisms by which heat stress can prevent the growth of oocytes. The foremost is the reduction on the synthesis of preovulatory surge in luteinizing hormone and estradiol. Hence, there is poor follicle maturation and this leads to ovarian inactivity in cattle [46]. Heat stress also delays follicle selection and reduces the degree of dominance of the dominant follicle. Heat stress decreases blood progesterone concentration, which is a major cause for abnormal oocyte maturation, implantation failure and finally early embryonic death in dairy cattle [47]. During heat stress, the intrauterine environment of the cow is compromised. Hence, there is decrease in blood flow to the uterus and elevated uterine temperature. These changes increase the chances of early embryonic loss and suppress embryonic development [48]. The exposure of females to heat stress conditions during days 0-3 of pregnancy or days 0-7 of pregnancy reduced embryonic survival. Heat stress has a deleterious effect on the oocyte quality in buffaloes. Follicular growth and atresia during anestrus are attributed to the inadequate secretion of gonadotropins by the hypophysis [18]. There is decrease in the concentration of oestradiol-17 beta in summer which reduces the intensity of estrus manifestation and results in silent heat in buffaloes [40]. The mean plasma prolactin concentration was significantly higher in summer than winter which may cause acyclicity or infertility in buffaloes [49].

## Mitigation Strategies to Combat Heat Stress

There is huge economic loss due to heat stress in livestock. In India, there loss of 1.8 million tonnes of milk a year due to heat stress among cattle and buffaloes, which is attributable to approximately Rs. 2661 crore [50]. Poor nutrition, inappropriate management and environmental factors have a significant negative influence on reproductive efficiency of cattle [51,52]. Basically three mitigation strategies are applied to combat the negative influences of heat stress on animals which are described in the followings:

- Development of genetically heat tolerant dairy breeds
- Nutrition modification
- Environment modification
- Timed artificial insemination (TAI) protocol.

### Development of genetically heat tolerant dairy breeds

Selection for higher milk yield in cows has led to increased metabolic heat production and this causes more susceptibility of the animal towards heat stress. The productivity and heat tolerance are antagonistic. The continual selection for production ignoring heat tolerance results in decreasing heat tolerance. There is the presence of considerable variation for heat tolerance between breeds and even between individuals within a breed. The identification and selection of heat tolerant dairy animals is useful to maintain both the high productivity and survivability when exposed to heat stress conditions. Therefore, the inclusion of THI covariate effects in the selection index should be targeted for genetic evaluation of dairy animals especially in the hot climate [53].

Improving animal adaptation to climate stress can be achieved in two ways such as through selection of the animals in heat stressed conditions and through introgressing heat adaptation genes from a local breed into a commercial herd [54]. The genes responsible for traits like coat color and hair length confer heat shock resistance in cells [55]. Hair coat characteristics like hair coat thickness and hair weight per unit surface are important determinant of non-evaporative heat loss from the body. The slick hair gene has been identified for increased thermal resistance due to its association with increased sweating rate and a lower metabolic rate in animals [56]. The slick hair gene responsible for slick hair coat improves heat tolerance capacity when introduced into temperate climate cattle breeds [57]. There are some other genes *viz*., ATP1A1 gene and heat shock protein (HSP) genes have been identified which confer thermal resistance and adaptation to thermal stress in cattle [58,59]. The ATP1A1 gene is known as Na+/K+ -ATPase subunit alpha-1. This gene is well recognized as a candidate for heat shock response because of its association to oxidative stress in cattle [58]. The bovine Na+/K+ -ATPase protein complex consists of α and β subunits. The ATP1A1 gene encodes the α1 isoform, the major isoform of α subunit of Na^+^-K^+^ ATPase pump. ATP1A1 gene has been mapped on *B. taurus* chromosome number 3 and is consisting of 22 introns, 23 exons. ATP1A1 gene is responsible for establishing the electrochemical gradient of Na+ and K+ across the plasma membrane, which is essential in the maintenance of body fluid and cellular homeostasis. The ATPA1 gene, ATPB2 gene and osteopontin were found to have significant association with thermo-tolerance in buffaloes [60]. The cellular heat shock response is another component of adaptation to heat stress. During hyperthermia, heat stress activates heat shock transcription factor-1 and this enhances expression of HSPs coupled with decreased expression and synthesis of other proteins, and HSP induced activation of immune system. The role of HSP is to activate the immune and endocrine system and also to alter the physiological state referred to as acclimation. The detailed understanding of genes in regulating the heat shock response in animals would be helpful to improve their thermal tolerance via gene manipulation [61]. The HSP70 family genes were found highly expressed in summer in Sahiwal and Tharparkar cattle and buffalo in India which enhances their thermotolerance and adaptive capacity to dry/hot humid environment [62].

The identification of major genes associated with thermo-tolerance that reduces the effects of heat stress in cattle and buffaloes and its subsequent incorporation into breeding program through marker assisted selection should be the breeding strategy for enhancing both the reproductive ability and adaptability to the warm climate. The crossbred cattle are better adapted and have better reproductive performance than purebred exotic cattle [63]. The thermal tolerance and reproductive performances of exotic cattle breeds can be improved by crossbreeding them with local cattle breeds.

### Nutrition modification

When the cows are under heat stress, there is decrease in dry matter intake along with crude protein intake and due to reduced feed intake, there is negative energy balance in the body of heat stressed cows and buffaloes. Due to increase in core body temperature and inefficient heat dissipation processes, energy requirements for maintenance is found to be increased. Therefore, the measures to increase the nutrient density include feeding of high quality forage, concentrates and use of supplemental fats in the diet of animals. The feed additives are also very useful to stabilize the rumen environment from dietary modifications and also improve the energy utilization [64]. The dry matter digestibility and protein/energy ratio were also found to be decreased in heat stress conditions. Feeding of good quality low-degradable protein has shown to improve milk production in heat stressed cows. So both quantity and form of protein play important role during feeding of the heat stressed cows and buffaloes. Feeding supplemental niacin is also helpful in reducing the effects of heat stress in cattle. Supplementation with antioxidants during the heat stress period is another way to improve fertility through a decrease of oxidative stress in buffaloes [65].

### Environment modification

The months of May and June were found very critical for the optimum reproductive performances in dairy animals. There was reduction in breeding values of reproductive traits in buffaloes during the CHSZ of May and June [66]. Therefore, in order to prevent the effects of heat stress, the modification of the surrounding environment is the key management practices to be followed in the dairy herd. Primary methods for altering the environment can be classified into two categories; first is the provision of shade and the other is evaporative cooling strategies with water. Provision of shade protects the cows and buffaloes from direct effects of solar radiation. Trees are an excellent source of shade combined with beneficial cooling as moisture evaporates from the leaves and animals also preferentially seek for tree shade over artificial shade structures [28]. Artificial shades are quite useful for heat stressed animals in confinement or in intensive situations. Shade is effective in protecting cows from solar radiation, but does not alter the air temperature or RH around the cows.

Though the evaporative cooling strategies are costly, but they are more useful to alleviate the heat stress in animals. Evaporative cooling systems use the energy from the air to evaporate water and evaporation of water into warm air reduces the air temperature. The milk production and reproductive performances of dairy cattle were improved using an evaporative cooling system [67]. Fogging systems use very fine droplets of water and these water droplets are immediately dispersed into the air stream and quickly evaporate, thus cooling the surrounding air. Misting systems generate larger droplets than fogging systems, but cool the air by the same principle. The sprinklers are different from foggers and misters. The sprinklers do not cool the air rather than the large droplet arising from them wet the hair coat and skin of the cows and buffaloes and then water evaporates to cool the hair and skin. This system is a very effective in combination with air movement. The mechanical air cooling is possible by using the evaporative cooling pad and fan system which are very useful in reducing the rectal temperature and respiratory rate in cows and buffaloes.

### Timed artificial insemination (TAI) protocol

Heat stress reduces the length and intensity of estrus and hence the incidences of anestrous and silent ovulation are increased. The use of TAI protocol is practiced for accurate estrus detection and timely insemination in order to improve fertility in summer. Hormonal treatments have been developed to synchronize the time of ovulation, allowing the use of fixed TAI that does not require detection of estrus. The TAI protocol is commonly referred to as ovsynch and this consists of hormonal treatments of gonadotropin-releasing hormone (GnRH) (day 0), prostaglandin F2α (day 7) and GnRH (day 9) and artificial insemination is being performed 16-20 h after second GnRH treatment [68]. The ovysynch protocol can successfully synchronize ovulation in buffaloes and can also increase conception rate when combined with TAI [69]. The CIDRsynch and Presynch protocols are also applied to improve conception rate and pregnancy rate of Holstein cows under subtropical environmental conditions [70]. This TAI protocol can be able to reduce losses in reproductive efficiency in cattle and buffaloes caused by poor estrus detection in summer.

## Conclusion

The heat stress has adverse effects on the reproductive performances of cattle and buffaloes. The THI is the most commonly used index to measure the level of heat stress in animals. The reproductive traits of cattle are susceptible to the negative impacts of heat stress with increase in THI above 72, while the buffaloes are more prone to heat stress when the THI level surpasses 75. The months from April to September under HSZ show the average THI level above 75. This necessitates the adoption of proper management interventions in the form of nutrition modification and environment modification in order to ameliorate the effects of heat stress on cattle and buffaloes during April to September. The heat tolerant dairy animals should be selected which can enhance both the reproductive ability and adaptability to the warm climate.

## Authors’ Contributions

SD prepared the initial version of the manuscript. AU, MS and SY assisted in literature collection. SD, AKC and AS revised the manuscript and made final critical scientific corrections. All authors read and approved the final manuscript.

## References

[1] BAHS, Basic Animal Husbandry Statistics (2014). Department of Animal Husbandry, Dairying and Fisheries, Ministry of Agriculture, Government of India.

[2] NDRI Vision 2030

[3] Thiruvenkadan A.K, Panneerselvam S, Rajendran R, Murali N (2010). Analysis on the productive and reproductive traits of Murrah buffalo cows maintained in the coastal region of India. Appl. Anim. Husb. Rural Dev.

[4] Hahn G.L, Mader T.L, Eigenberg R.A (2003). Perspectives on development of thermal indices for animal studies and management. Proceeding Symposium. Interactions between Climate and Animal Production. EAAP Technical Series No. 7.

[5] Buffington D.E, Collazo-Arochu A, Canton H.H, Pritt D, Thatcher W.W, Collier R.J (1981). Black globe-humidity index (BGHI) as comfort equation for cows. Trans. Am. Soc. Agric. Eng.

[6] Ravagnolo O, Misztal I (2002). Effect of heat stress on non return rate in Holsteins: Fixed-model analyses. J. Dairy Sci.

[7] Garcia-Ispierto I, Lopez-Gatius F, Bech-Sabat G, Santolaria P, Yaniz J.L, Nogareda C, De Rensis F, Lopez-Bejar M (2007). Climate factors affecting conception rate of high producing dairy cows in northeastern Spain. Theriogenology.

[8] Dash S, Chakravarty A.K, Sah V, Jamuna V, Behera R, Kashyap N, Deshmukh B (2015). Influence of temperature and humidity on pregnancy rate of Murrah buffaloes. Asian-Aust. J. Anim. Sci.

[9] Thom E.C (1959). The discomfort index. Weatherwise.

[10] Gaughan J.B, Mader T.L, Holt S.M, Lisle A (2008). A new heat load index for feedlot cattle. J. Anim. Sci.

[11] Morton J.M, Tranter W.P, Mayer D.G, Jonsson N.N (2007). Effect of environmental heat on conception rates in lactating dairy cows: Critical periods of exposure. J. Dairy Sci.

[12] Schuller L.K, Burfeind O, Heuwieser W (2014). Impact of heat stress on conception rate of dairy cows in the moderate climate considering different temperature humidity index thresholds, periods relative to breeding, and heat load indices. Theriogenology.

[13] Dash S (2013). Genetic evaluation of fertility traits in relation to heat stress in Murrah buffaloes.

[14] McGowan M.R, Mayer D.G, Tranter W, Shaw M, Smith C, Davison T.M (1996). Relationship between temperature humidity index and conception efficiency of dairy cattle in Queensland. Proc. Aust. Soc. Anim. Prod.

[15] Khan F.A, Prasad S, Gupta H.P (2013). Effect of heat stress on pregnancy rates of crossbred dairy cattle in Terai region of Uttarakhand, India. Asian Pac. J. Reprod.

[16] IPCC, (Intergovernmental Panel on Climate Change) (2014). Climate Change: Synthesis Report; Summary for Policymakers.

[17] Singh G, Totey S.M, Talwar G.P (1989). *In vitro* fertilization of buffalo (*Bubalus bubalis*) oocytes matured *in vitro*. Theriogenology.

[18] Das G.K, Khan F.A (2010). Summer anoestrus in buffalo - A review. Reprod. Domest. Anim.

[19] Upadhyay R.C, Rita Rani A, Singh S.V, Mohanty T.K, Gohain M (2012). Impact of climate change on reproductive functions of Murrah buffaloes. J. Anim. Plant Sci.

[20] National Research Council (1971). A Guide to Environmental Research on Animals.

[21] Yousef M.K (1985). Stress Physiology in Livestock.

[22] Bianca W (1962). Relative importance of dry- and wet-bulb temperatures in causing heat stress in cattle. Nature.

[23] Mader T.L, Davis M.S, Brown-Brandl T (2006). Environmental factors influencing heat stress in feedlot cattle. J. Anim. Sci.

[24] Dash S, Chakravarty A.K, Singh A, Sah V, Shivahre P.R, Panmei A (2015). Identification of best temperature humidity index model for pregnancy rate of Murrah buffaloes in a subtropical climate. Indian J. Dairy Sci.

[25] Bohmanova J, Misztal I, Cole J.B (2007). Temperature-humidity indices as indicators of milk production losses due to heat stress. J. Dairy Sci.

[26] Berman A, Folman Y.M, Kaim M, Mamen Z, Herz D, Wolfenson A, Graber Y (1985). Upper critical temperatures and forced ventilation effects for high-yielding dairy cows in a tropical climate. J. Dairy Sci.

[27] Armstrong D.V (1994). Heat stress interactions with shade and cooling. J. Dairy Sci.

[28] McDowell R.E, Hooven N.W, Camoens J.K (1976). Effects of climate on performance of Holsteins in first lactation. J. Dairy Sci.

[29] Moran J (2005). Tropical Dairy Farming: Feeding Management for Small Holder Dairy Farms in the Humid Tropics.

[30] Kadzere C.T, Murphy M.R, Silanikove N, Maltz E (2002). Heat stress in lactating dairy cows: A review. Livest. Prod. Sci.

[31] Dash S, Chakravarty A.K, Singh A, Behera R, Upadhyay A, Shivahre P.R (2014). Determination of critical heat stress zone for fertility traits using temperature humidity index in Murrah buffaloes. Indian J. Anim. Sci.

[32] Kaewlamun W, Chayaratanasin R, Virakul P, Andrew A.P, Humblot P, Suadsong S, Tummaruk P, Techakumphu M (2011). Differences of periods of calving on days open of dairy cows in different regions and months of Thailand. Thai J. Vet. Med.

[33] Boonkum W, Misztal I, Duangjinda M, Pattarajinda V, Tumwasorn S, Buaban S (2011). Genetic effects of heat stress on days open for Thai Holstein crossbreds. J. Dairy Sci.

[34] Kumar A, Gandhi R.S (2011). Evaluation of pooled lactation production and reproduction traits in Sahiwal cattle. Indian J. Anim. Sci.

[35] Wolfenson D, Roth Z, Meidan R (2000). Impaired reproduction in heat-stressed cattle: Basic and applied aspects. Anim. Reprod. Sci.

[36] Oseni S, Mistzal I, Tsuruta S, Rekaya R (2004). Genetic components of days open under heat stress. J. Dairy Sci.

[37] Nabenishi H, Ohta H, Nishimoto T, Morita T, Ashizawa K, Tsuzuki Y (2011). Effect of the temperature humidity index on body temperature and conception rate of lactating dairy cows in southwestern Japan. J. Reprod. Dev.

[38] Zicarelli L (2010). Enhancing reproductive performance in domestic dairy water buffalo (*Bubalus bubalis*). Soc. Reprod. Fertil. Suppl.

[39] Abayawansa W.D, Prabhakar S, Singh A.K, Brar P.S (2011). Effect of climatic changes on reproductive performance of Murrah buffaloes in Punjab: A retrospective analysis. Indian J. Anim. Sci.

[40] Singh M, Chaudhari B.K, Singh J.K, Singh A.K, Maurya P.K (2013). Effects of thermal load on buffalo reproductive performance during summer season. J. Biol. Sci.

[41] El-Wishy A.B (2013). Fertility of Holstein cattle in a subtropical climate of Egypt. Iran. J. Appl. Anim. Sci.

[42] El-Tarabany M.S, El-Bayoumi K.M (2015). Reproductive performance of backcross Holstein x Brown Swiss and their Holstein contemporaries under subtropical environmental conditions. Theriogenology.

[43] Oseni S, Misztal I, Tsuruta S (2005). Genetic parameters for pregnancy rate in Holstein cattle under seasonal heat stress. Nig. J. Genet.

[44] Amundson J.L, Mader T.L, Rasby R.J, Hu Q.S (2006). Environmental effects on pregnancy rate in beef cattle. J. Anim. Sci.

[45] Vale W.G (2007). Effects of environment on buffalo reproduction. Ital. J. Anim. Sci.

[46] Hansen P.J (2007). Exploitation of genetic and physiological determinants of embryonic resistance to elevated temperature to improve embryonic survival in dairy cattle during heat stress. Theriogenology.

[47] Khodaei-Motlagh M, Shahneh A.Z, Masoumi R, Derensis F (2011). Alterations in reproductive hormones during heat stress in dairy cattle. Afr. J. Biotechnol.

[48] De Rensis F, Scaramuzzi R.J (2003). Heat stress and seasonal effects on reproduction in the dairy cow: A review. Theriogenology.

[49] Roy K.S, Prakash B.S (2007). Seasonal variation and circadian rhythmicity of the prolactin profile during the summer months in repeat-breeding Murrah buffalo heifers. Reprod. Fertil. Dev.

[50] Upadhayay R.C (2010). Annual Milk Production Loss Due to Global Warming.

[51] Fair T (2010). Mammalian oocyte development: Checkpoints for competence. Reprod. Fertil. Dev.

[52] Walsh S.W, Williams E.J, Evans A.C.O (2011). A review of the causes of poor fertility in high milk producing dairy cows. Anim. Reprod. Sci.

[53] Bernabucci U, Biffani S, Buggiotti L, Vitali A, Lacetera N, Nardone A (2014). The effects of heat stress in Italian Holstein dairy cattle. J. Dairy Sci.

[54] Renaudeau D, Collin A, Yahav S, de Basilio V, Gourdine J.L, Collier R.J (2012). Adaptation to hot climate and strategies to alleviate heat stress in livestock production. Animal.

[55] Hansen P.J, Arechiga C.F (1999). Strategies for managing reproduction in the heat stressed dairy cow. J. Anim. Sci.

[56] Dikmen S, Alava E, Pontes E, Fear J.M, Dikmen B.Y, Olson T.A, Hansen P.J (2008). Differences in thermoregulatory ability between slick-haired and wild-type lactating Holstein cows in response to acute heat stress. J. Dairy Sci.

[57] Berman A (2011). Invited review: Are adaptations present to support dairy cattle productivity in warm climates?. J. Dairy Sci.

[58] Liu Y.X, Zhou X, Li D.Q, Cui Q.W, Wang G.L (2010). Association of ATP1A1 gene polymorphism with heat tolerance traits in dairy cattle. Genet. Mol. Res.

[59] Loredana B, Patrizia M, Valentina P, Nicola L, Alessandro N, Umberto B (2011). Cellular thermotolerance is associated with heat shock protein 70.1 genetic polymorphisms in Holstein lactating cows. Cell Stress Chaperones.

[60] Jayakumar S (2014). Molecular characterization of thermoregulatory genes in Murrah buffaloes.

[61] Collier R.J, Collier J.L, Rhoads R.P, Baumgard L.H (2008). Genes involved in the bovine heat stress response. J. Dairy. Sci.

[62] Kumar A, Ashraf S, Goud T.S, Grewal A, Singh S.V, Yadav B.R, Upadhyay R.C (2015). Expression profiling of major heat shock protein genes during different seasons in cattle (*Bos indicus*) and buffalo (*Bubalus bubalis*) under tropical climatic condition. J. Therm. Biol.

[63] El-Tarabany M.S, Nasr M.A.F (2015). Reproductive performance of Brown Swiss, Holstein and their crosses under subtropical environmental conditions. Theriogenology.

[64] Zimbelman R.B, Baumgard L.H, Collier R.J (2010). Effect of encapsulated niacin on evaporative heat loss and body temperature in moderately heat-stressed lactating Holstein cows. J. Dairy Sci.

[65] Megahed G.A, Anwar M.M, Wasfy S.I, Hammadeh M.E (2008). Influence of heat stress on the cortisol and oxidant-antioxident balance during oestrous phase in buffalo-cows (*Bubalus bubalis*): Thermo-protective role of antioxidant treatment. Reprod. Domest. Anim.

[66] Dash S, Chakravarty A.K, Singh A, Shivahre P.R, Upadhyay A, Sah V, Singh K.M (2015). Assessment of expected breeding values for fertility traits of Murrah buffaloes under subtropical climate. Vet. World.

[67] Ryan D.P, Boland M.P, Kopel E, Armstrong D, Munyakazi L, Godke R.A, Ingraham R.H (1992). Evaluating two different evaporative cooling management systems for dairy cows in a hot, dry climate. J. Dairy Sci.

[68] Ambrose D.J, Colazo M.G, Kastelic J.P (2010). The applications of timed artificial insemination and timed embryo transfer in reproductive management of dairy cattle. R. Bras. Zootec.

[69] Hoque M.N, Talukder A.K, Akter M, Shamsuddin M (2014). Evaluation of ovsynch protocols for timed artificial insemination in water buffaloes in Bangladesh. Turk. J. Vet. Anim. Sci.

[70] El-Tarabany M.S, El-Tarabany A.A (2015). Impact of thermal stress on the efficiency of ovulation synchronization protocols in Holstein cows. Anim. Reprod. Sci.

